# Involvement of splenic iron accumulation in the development of nonalcoholic steatohepatitis in Tsumura Suzuki Obese Diabetes mice

**DOI:** 10.1038/srep22476

**Published:** 2016-03-02

**Authors:** Kazutoshi Murotomi, Shigeyuki Arai, Satoko Uchida, Shin Endo, Hitoshi Mitsuzumi, Yosuke Tabei, Yasukazu Yoshida, Yoshihiro Nakajima

**Affiliations:** 1 Health Research Institute, National Institute of Advanced Industrial Science and Technology (AIST), Takamatsu, Kagawa 761-0395, Japan; 2 Hayashibara Co., Ltd., Naka-ku, Okayama 702-8006, Japan

## Abstract

Nonalcoholic steatohepatitis (NASH) is a common hepatic manifestation of metabolic syndrome and can lead to hepatic cirrhosis and cancer. It is considered that NASH is caused by multiple parallel events, including abnormal lipid metabolism, gut-derived-endotoxin-induced inflammation, and adipocytokines derived from adipose tissue, suggesting that other tissues are involved in NASH development. Previous studies demonstrated that spleen enlargement is observed during the course of NASH pathogenesis. However, the involvement of splenic status in the progression of NASH remains unclear. In this study, we examined hepatic and splenic histopathological findings in the early stage of NASH using the Tsumura Suzuki Obese Diabetes (TSOD) mouse model established for assessing NASH. We found that 12-week-old TSOD mice clearly exhibited the histopathological features of NASH in the early stage. At this age, the spleen of TSOD mice showed markedly higher iron level than that of control Tsumura Suzuki Non Obesity (TSNO) mice. The level of accumulated iron was significantly decreased by feeding a diet with glucosyl hesperidin, a bioactive flavonoid, accompanied with alleviation of hepatic lesions. Furthermore, we found that splenic iron level was positively correlated with the severity of NASH manifestations, suggesting that abnormalities in the spleen are involved in the development of NASH.

Nonalcoholic fatty liver disease (NAFLD) is one of the common hepatic manifestations of metabolic syndrome, and its prevalence is rapidly increasing worldwide^1^. A certain percentage of patients with NAFLD develop nonalcoholic steatohepatitis (NASH), which is characterized by steatosis, lobular inflammation, and fibrosis in the liver, and can lead to serious and irreversible diseases, including hepatic cirrhosis and cancer^2^. The biopsy of the liver from NAFLD/NASH patients shows the typical features of hepatocellular ballooning, pigmented macrophages, and glycogenated nuclei^3^. The mechanism underlying the development of NASH has been based on the concept of the “two-hits hypothesis”^4^. The first hit is hepatic steatosis defined as the accumulation of hepatic triglycerides (TGs) in more than 5% of hepatocytes^5^ mediated by obesity and insulin resistance, and the second hit is the production of free radicals and inflammatory cytokines. Recently, the “multiple-parallel-hits hypothesis” has been proposed as the more logical mechanism, by which the development of NASH is associated with abnormal lipid metabolism, oxidative stress, adipocytokines, and gut-derived-endotoxin-induced inflammation, mitochondrial dysfunction, and endoplasmic reticulum (ER) stress^6^,^7^,^8^. The adipocytokines, leptin^9^ and adiponectin^10^ derived from adipose tissue and gut-derived endotoxins^8^ induce hepatic lipid accumulation and fibrosis as NAFLD/NASH progresses. Although these findings suggest that not only the liver per se but also other tissues also participate in the development of NASH, the relationship between the severity of NASH and the state of other tissues is unknown.

The spleen, which plays a critical role in the modulation of the immune system, is anatomically linked to the liver, and splenic volume increases according to the severity of the compromised liver function. Thus, it has been considered that spleen enlargement (splenomegaly) is caused by liver congestion accompanied by decreased liver function^11^. A previous study indicated that the splenic volume in NAFLD patients is larger than that in normal subjects^12^. Recently, it has been indicated that the ultrasonographic spleen longitudinal diameter (SLD) in the NASH patient group is larger than that in the control group and that SLD is an effective marker to discriminate between simple steatosis and NASH with mild fibrosis^13^. Further confirmation of these findings by another study showed that the spleen volume in NASH patients with mild fibrosis is significantly larger than that in simple steatosis subjects, indicating spleen enlargement may be a distinct feature in early-stage NASH^14^. However, the significance of splenic status in relation with the progression of NASH in the early stage remains unclear.

Tsumura Suzuki Obese Diabetes (TSOD) mice have been established as a model of obese type 2 diabetes. Recently, it has been demonstrated that TSOD mice show the histopathological features of NASH, including microvesicular fatty degeneration, hepatocellular ballooning degeneration, Mallory bodies, and inflammatory cell infiltration from 4 months of age, and liver tumors are observed in the mouse at 12 months of age^15^. Thus, the TSOD mouse model is considered useful for assessing NAFLD/NASH caused by metabolic syndrome^15^.

In the present study, to clarify the relationship between the severity of NASH and the splenic status, we investigated the histopathological findings of the liver and spleen in the early stage of NASH using TSOD mice. At the age of 12 weeks, iron markedly accumulated in the spleen of TSOD mice compared with control Tsumura Suzuki Non Obesity (TSNO) mice. Hepatic lesion formation and splenic iron overload in TSOD were attenuated by the feeding of a diet supplemented with a flavonoid, glucosyl hesperidin (Ghes), which is a glucosyl derivative of hesperidin, which has a liver protective effect against lipid metabolism abnormalities^16^. This finding suggests that accumulation of splenic iron may synergistically occur during the progression of NASH. In addition, we found that splenic iron level positively correlated with the severity of NASH manifestation. Our findings suggest that the abnormalities in the spleen, such as iron overload, are involved in the pathogenesis and progression of early-stage NASH.

## Results

### Histopathological observations of liver

**Figure 1:**
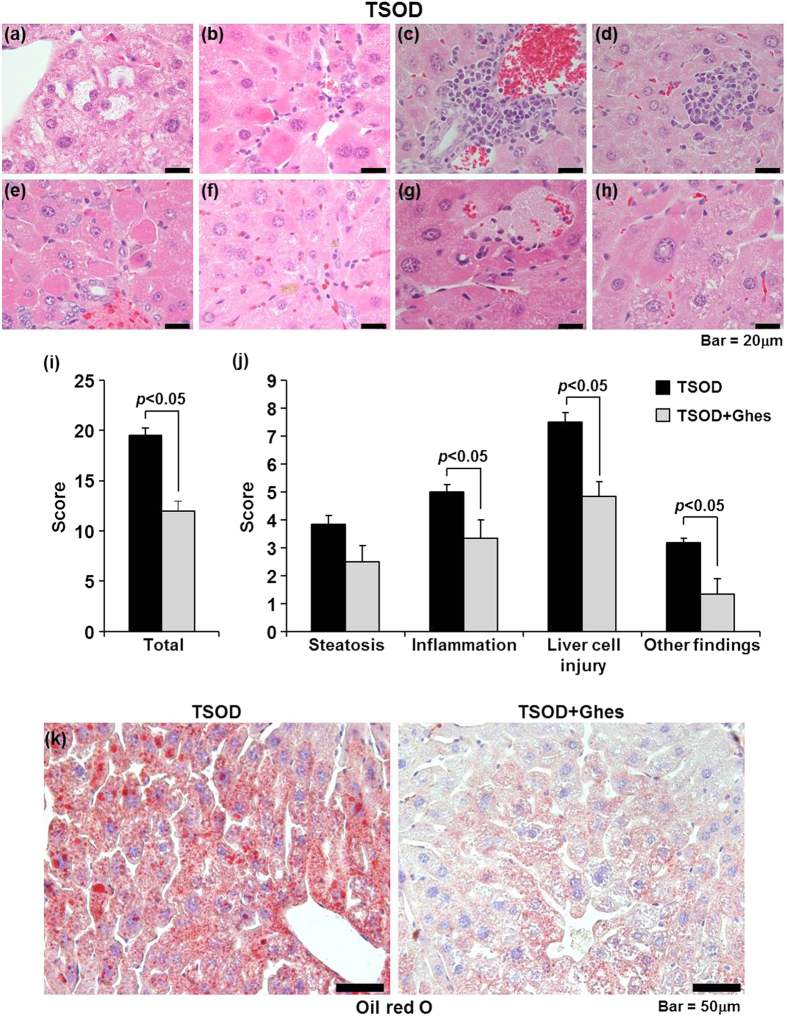
Characteristic liver histopathological findings of initial NASH changes in TSOD mouse at 12 weeks of age. High magnification of liver lesions in TSOD mouse (**a–h**; 1000× magnification). Representative findings of NASH, including ballooning (vacuolar) degeneration (**a**), eosinophilic and Mallory-like bodies (**b**), portal inflammation (**c**), lobular microgranuloma (**d**), hyalinization and cytoplasmic eosinophilic droplets (large mitochondria) of hepatic cells (**e**), pigmented macrophages and acidophil bodies (**f**), autolysis of hepatic cells, hemorrhage and inflammatory cell infiltration (**g**), and glycogenated nuclear cells (**h**), were observed in TSOD mice. Liver histopathological features were scored according to the modified method of NASH Clinical Research Network Scoring System (**i,j**). Results are expressed as means ± standard error (n = 6 each). Statistical analyses of total score and other items were carried out using ANOVA (Fisher’s PLSD test) and Mann-Whitney U-test, respectively. Oil-red-O-stained frozen liver sections from control-diet-fed TSOD and Ghes-fed TSOD mice (**k**) at 12 weeks of age (400× magnification).

A previous study indicated that TSOD mice show the histopathological features of NASH from 4 months (calc: 16 to 20 weeks) of age^15^. In addition, we previously showed that at around 12 weeks of age, TSOD mice develop obesity and insulin resistance, which are the major risk factors for the development of NASH, compared with control TSNO mice, suggesting that TSOD mice exhibit the phenotypes in the early stage of NASH. In order to investigate the hepatic status in TSOD mice, we measured the serum biochemical parameters used in the liver function test. As shown in Supplementary Fig. S1, the levels of serum aspartate aminotransferase (AST), alanine aminotransferase (ALT), total cholesterol, and TG in 12-week-old TSOD mice were significantly higher than those in age-matched TSNO mice, suggesting that the liver in TSOD mice was abnormal. Next, we analyzed the histopathological features of the liver of TSNO and TSOD mice. Cytoplasmic vacuolar degeneration of liver cells was clearly observed in the hepatic lobule of TSOD mice at 12 weeks of age compared with that of age-matched TSNO mice (Supplementary Fig. S2). In addition, TSOD mice exhibited other characteristic findings of NASH, including ballooning degeneration (Fig. 1a), Mallory-like bodies (Fig. 1b), infiltration of mononuclear cells (Fig. 1c), lobular microgranuloma (Fig. 1d), hyaline droplets of hepatocytes (Fig. 1e), pigmented macrophages and acidophil bodies (Fig. 1f), autolysis of hepatocytes (Fig. 1g), and glycogenated nuclear cells (Fig. 1h). On the other hand, no liver fibrosis was observed in TSOD mice at 12 weeks of age. These findings clearly indicated that TSOD mice at this age exhibit the histopathological features of NASH in the early stage.

To quantify the severity of NASH, we scored the pathological condition of the liver according to the modified version of the method proposed by the NASH Clinical Research Network^3^ (Supplementary Table 1). The total score of TSOD mice was 19.5 ± 0.72 (maximum score, 45) (Fig. 1i; black bars), whereas that of TSNO mice was zero. Following the feeding of the diet supplemented with Ghes, which improves the liver function against hypertriglyceridemia^17^, the total score and the score of each of the items, including inflammation and liver cell injury, of TSOD mice significantly decreased (Fig. 1i, j; gray bars).

Next, we investigated hepatic lipid accumulation in TSOD mice. As shown in Supplementary Fig. S3a, there were no significant differences in liver TG levels among TSNO, control-diet-fed TSOD, and Ghes-supplemented-diet-fed (hereafter, Ghes-fed) TSOD mice. The liver cholesterol levels were not also significantly different between the TSNO and TSOD mice. The feeding of the Ghes-supplemented diet significantly decreased the liver cholesterol level (Supplementary Fig. S3b), whereas that of Ghes had no effect on serum ALT, AST, TG, and cholesterol levels in TSOD mice (Supplementary Fig. S1). On the other hand, lipid accumulation was clearly observed in liver zone 3 of the control-diet-fed TSOD mice (Fig. 1k, left panel), and lipid accumulation was attenuated in the Ghes-fed TSOD mice (Fig. 1k, right panel). These findings indicate that the TSOD mouse model is suitable for analyzing the pathological conditions in the early stage of NASH and that Ghes is useful for alleviating the initial manifestations of NASH. Our findings further suggest that this mouse model and Ghes are useful tools for analyzing the relationship between the formation of hepatic lesions and the splenic status in the early stage of NASH.

### Analysis of hepatic iron levels

**Figure 2:**
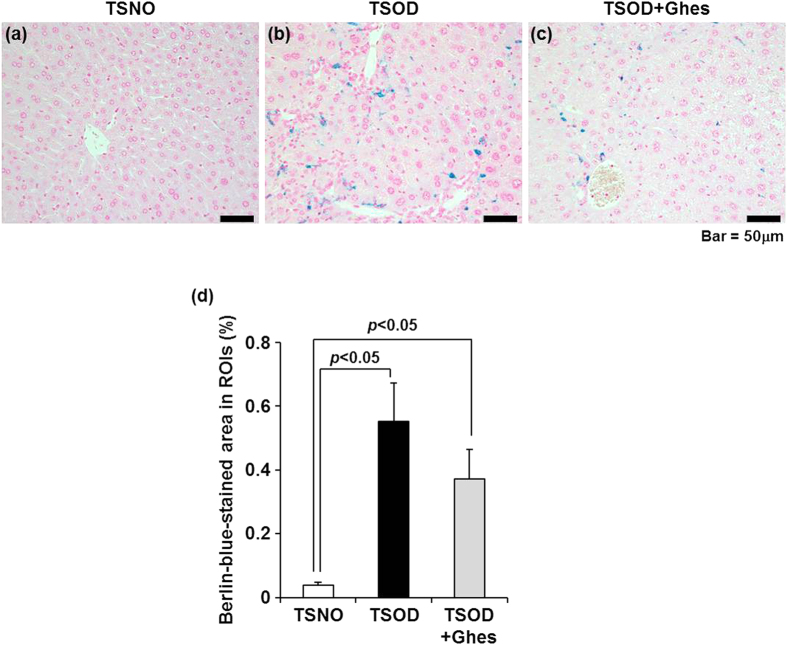
Hepatic iron deposition in TSOD mouse. Representative findings of Berlin-blue-stained liver sections from TSNO (**a**), control-diet-fed TSOD (**b**), and Ghes-fed TSOD (**c**) mice (400× magnification). The ratio of Berlin-blue-stained area was calculated as the average of the ratios of Berlin-blue-stained area to ROI area in five regions (**d**). Results are expressed as means ± standard error (n = 6 each). Statistical analyses were carried out using ANOVA (Fisher’s PLSD test).

Previous studies indicated that iron overload, which induces oxidative stress via the acceleration of Fenton reaction, is observed in the liver of NAFLD/NASH patients^18^,^19^. In addition, it was indicated that the hepatic iron deposition pattern is associated with the histopathological severity in the liver^18^. We, therefore, investigated whether hepatic iron overload is observed in TSOD mice at 12 weeks of age by histopathological analysis with Berlin blue staining. The liver from the control TSNO mice showed slight blue staining (Fig. 2a), whereas that from the TSOD mice clearly showed blue staining (Fig. 2b). In the quantification of the staining, the Berlin-blue-stained area in the control-diet-fed TSOD mice was significantly larger than that in the TSNO mice (Fig. 2d). In the comparison between the control-diet-fed and Ghes-fed TSOD mice, the Berlin-blue-stained area in the TSOD mice tended to be decreased by feeding of the Ghes-supplemented diet (Fig. 2c,d).

### Histopathological analysis of spleen

**Figure 3:**
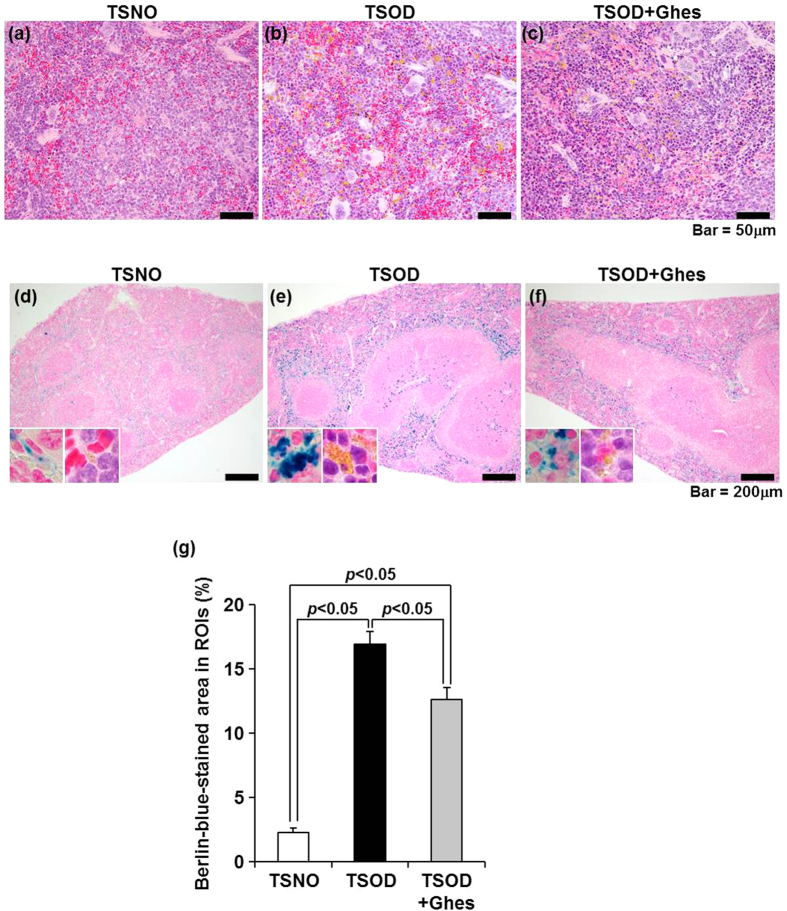
Histopathological findings of spleen in TSOD mouse. Representative findings of H&E-stained spleen sections from TSNO (**a**), control-diet-fed TSOD (**b**), and Ghes-fed TSOD (**c**) mice (400× magnification). Representative findings of Berlin blue-stained spleen sections from TSNO (**d**), control-diet-fed TSOD (**e**), and Ghes-fed TSOD (**f**) mice (100× magnification). The insets are enlarged images of Berlin-blue- and H&E-stained splenic red pulp. The ratio of Berlin-blue-stained area was calculated as the average of the ratios of Berlin blue-stained area to ROI area in five regions (**g**). Results are expressed as means ± standard error (n = 6 each). Statistical analyses were carried out using ANOVA (Fisher’s PLSD test).

To analyze the splenic status in the early stage of NASH, we conducted histopathological analysis to determine whether splenic damage occurs in 12-week-old TSOD mice. Hematoxylin and eosin (H&E) staining showed no marked histopathological changes, including splenic cell injury, in TSOD mice compared with the TSNO mice (Fig. 3a,b). However, obvious deposition of hemosiderin, which is the storage form of recycled iron in macrophages, in spleen was observed in TSOD mice (Fig. 3b). Therefore, we investigated the splenic iron level in TSOD mice by both inductively coupled plasma-mass spectrometry (ICP-MS) and Berlin blue staining. As shown in Supplementary Fig. S4, the splenic iron levels measured by ICP-MS in TSOD mice at 5 and 12 weeks of age were significantly higher than those in age-matched TSNO mice, whereas no significant differences were observed in serum iron levels between TSNO and TSOD mice at 12 weeks of age (Supplementary Fig. S5). Although the splenic iron level in TSOD mice at 5 weeks of age was 4.1-fold higher than that of age-matched TSNO mice, that in TSOD mice at 12 weeks of age was 42.6-fold higher, suggesting that the splenic iron levels in TSOD mice markedly increased during the pathogenesis of NASH (Supplementary Fig. S4). The Berlin-blue-stained area in the spleen almost overlapped with the H&E-stained brown granules of hemosiderin (Fig. 3d–f, enlarged images). Whereas the TSNO mice spleen showed weak Berlin blue staining (Fig. 3d), the TSOD mice clearly showed strong staining (Fig. 3e). In the quantification of the Berlin blue staining, the splenic Berlin-blue-stained area in the TSOD mice was significantly larger than that in TSNO mice (Fig. 3g). Berlin-blue-stained area in the Ghes-fed TSOD mice was significantly smaller than that in the control-diet-fed TSOD mice (Fig. 3c,f,g), which may be due to the attenuation of hepatic lesions by Ghes. These findings indicate that the TSOD mice showed marked iron accumulation in the spleen, as in the liver, in the early stage of NASH.

### Relationship between splenic iron levels and pathological manifestations of NASH in early-stage TSOD mice

**Figure 4:**
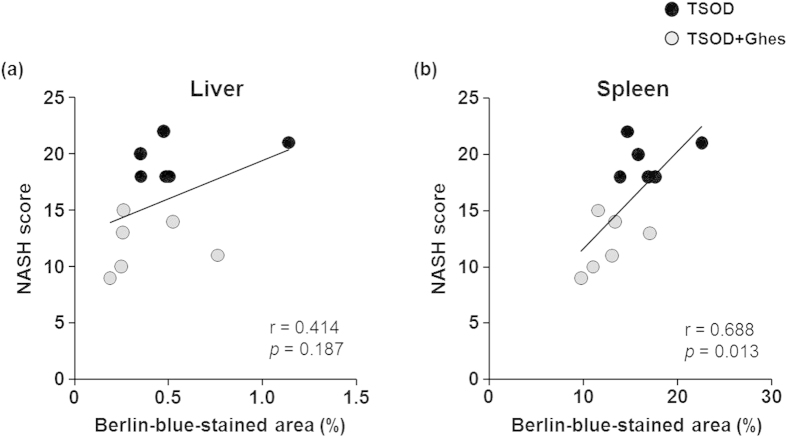
Correlation analysis between splenic iron level and NASH score in TSOD mice. Values of Berlin-blue-stained area in liver (**a**) and spleen (**b**) and NASH score for individual TSOD mice at 12 weeks of age. The strength of the association between two parameters was evaluated on the basis of Pearson’s correlation coefficient.

We analyzed the correlation between NASH score (Fig. 1i) and Berlin-blue-stained area in the liver (Fig. 2d) and spleen (Fig. 3g) from the TSOD mice. Although Berlin-blue-stained area in the liver moderately correlated with NASH score in the TSOD mice at 12 weeks of age, the correlation was not significant (Fig. 4a, *p* = 0.187). On the other hand, Berlin-blue-stained area in the spleen strongly correlated with NASH score in the TSOD mice, which was statistically significant (Fig. 4b, *p* = 0.013). These findings indicate that splenic iron level more accurately reflects the severity of NASH in the early stage than hepatic iron level, and that the alleviation of the hepatic lesions in the liver by feeding of a Ghes-supplemented diet is strongly related to splenic iron levels.

## Discussion

In this study, we found that the TSOD mice showed marked iron deposition in the spleen in the early stage of NASH (Fig. 3). It is notable that the severity of NASH significantly correlated with the iron levels in, not the liver, but the spleen (Fig. 4). An enhanced iron deposition induces oxidative^20^ and ER^21^ stresses, which are involved in the development of NASH. Free iron released from senescent erythrocytes phagocytosed by splenic macrophages forms a complex with transferrin, and the complex is transported to the liver via portal circulation. Excessive free iron in the spleen may contribute to the increase in hepatic iron level leading to oxidative stress. Therefore, it may be reasonable to assume that splenic iron overload induces the exacerbation of NASH through the portal circulation. It is noteworthy that iron locally accumulated in the spleen of TSOD mice from 5 to 12 weeks of age compared with that of TSNO mice without changes in serum iron level (Supplementary Figs S4 and S5). In addition, some reports indicated that spleen enlargement is observed in the pathogenesis of NASH^12^,^13^,^14^. Although it remains unclarified whether these events in the spleen are compensatory or pathological changes, the abnormalities in the spleen, such as iron overload, may play a key role in the development of NASH in the early stage.

It has been considered that progressive damage in the liver induces portal hypertension and splenic congestion^11^, which suggests accumulation of iron-containing erythrocytes. In addition, as numerous studies indicated that hesperidin has beneficial effects on various types of hepatic damage^17^,^22^,^23^, which is consistent with our results shown in Fig. 1, it was speculated that the alleviation of hepatic damage by Ghes mainly contributes to the suppression of the increase in the splenic iron level in TSOD mice. As the liver and spleen are strictly linked through circulation^24^, our results suggest that reciprocal events between the spleen and the liver play key roles in the development of pathological symptoms in the early stage of NASH.

Hepatic iron overload may be directly involved in the development of NASH, as dietary iron-loading aggravates steatohepatitis via hepatic cell injury^25^. However, our results indicated no correlation between hepatic iron level and NASH severity in TSOD mice at 12 weeks of age. A recent study found that predominant iron deposition in hepatocytes is associated with more severe liver damage, which is the increase in fibrosis stage to >1, in 587 Italian NAFLD patients^26^. As the 12-week-old TSOD mice showed negligible hepatic iron deposition (Fig. 3) and mild pathological condition of NASH (Fig. 1), more severe iron overload in hepatocytes, which could be observed in older TSOD mice, may contribute to the progression of NASH.

Functional ingredients, including polyphenols and flavonoids, are useful for the prevention and/or treatment of metabolic syndrome. It has been demonstrated that the treatment with Ghes reduced the serum TG level in hyperlipidemic subjects^17^. We, therefore, assumed that Ghes could effectively prevent the development of NAFLD/NASH. In this study, Ghes had no effects on the levels of serum AST, ALT, total cholesterol, and TG (Supplementary Fig. S1), and serum iron levels (Supplementary Fig. S5) in TSOD mice. However, from the scores determined using the modified NASH activity scoring system, the histopathological lesions, including inflammation, liver cell injury, and other findings, in TSOD mice were significantly reduced by Ghes treatment (Fig. 1i,j), without affecting body weight, food intake, and water consumption (Supplementary Fig. S6). It has been indicated that hesperidin chelates iron^27^ and protects the liver against various types of damage, including those induced by gamma-radiation^28^, carbon tetrachloride^29^, and high-cholesterol diet^30^. Although the mechanism underlying the preventive effect of Ghes against hepatic damage in NASH remains unclarified, various effects of Ghes may contribute to the attenuation of NASH symptoms. This study is the first to show the preventive effect of Ghes against the formation of hepatic lesions in the early stage of NASH.

In conclusion, we demonstrated that splenic iron accumulated in the early stage of NASH, and the iron levels significantly correlated with the severity of NASH. In addition, we found that Ghes prevented the splenic iron overload and the development of NASH in TSOD mice. Our results suggest that abnormal events in the spleen play a key role in the development of pathological symptoms in the early stage of NASH, and splenic iron level may be a suitable marker for evaluating the progression of NASH in the early stage.

## Methods

### Animals and experimental schedules

Four-week-old male TSOD mice and male TSNO mice (control) were obtained from the Institute for Animal Reproduction (Ibaraki, Japan). The animals were housed individually and had free access to a standard food (CE-2; Clea Japan Inc., Tokyo, Japan) and water. The animal room was maintained at 23 ± 2 °C and 50 ± 10% humidity under a 12 h light (8:00–20:00) and dark (20:00–8:00) cycle. The animals were acclimated to the laboratory environment for at least one week before the experiment. TSOD mice were divided into two weight-matched groups (n = 6 each); the control-diet-fed (CE-2) group and the group fed the diet supplemented with Ghes, which is a glucosyl derivative of hesperidin; the diet was synthesized with a higher water solubility of hesperidin by Hayashibara Co., Ltd. (Okayama, Japan). The Ghes-supplemented diet was prepared by mixing the control diet with 1.0% (w/w) Ghes and fed to the TSOD mice starting from 5 weeks of age. At 12 weeks of age, all the mice were euthanized under sevoflurane-induced anesthesia and the liver, spleen, and blood were collected for analysis. The animal experimental protocols were approved by the Institutional Animal Care and Use Committee of the National Institute of Advanced Industrial Science and Technology. All animal experiments were carried out in accordance with the approved protocols.

### Measurements of liver TG and cholesterol levels, body weight, food intake, and water consumption

To measure liver TG and cholesterol levels, the mice were sacrificed after 6 hours of fasting. Lipids were extracted from the liver by the Folch method. Briefly, the liver was homogenized with distilled water and suspended in chloroform/methanol (2 : 1) solution. After centrifugation at 3,500 rpm for 10 min, the lower layer was evaporated and reconstituted with isopropanol. Liver TG and cholesterol levels were measured with commercially available kits, Triglyceride E-test Wako and Cholesterol E-test Wako, respectively (Wako Pure Chemical Industries, Ltd., Osaka, Japan). Body weight, food intake, and water consumption were measured once a week.

### Measurements of serum biological parameters

After collecting blood from vena cava in mice, serum was harvested using CAPIJECT (Terumo Co., Ltd., Tokyo, Japan). Serum iron levels were measured using an iron assay kit Metalloassay according to the manufacturer’s instructions (Metallogenics Co., Ltd., Chiba, Japan). Other biological parameters, including AST, ALT, TG, and total cholesterol, in serum levels were measured with commercially available kits, Transaminase CII-test Wako, Triglyceride E-test Wako and Cholesterol E-test Wako, respectively (Wako Pure Chemical Industries, Ltd.).

### Histopathological analysis

The mouse liver and spleen were fixed in 10% buffered formalin and embedded in paraffin. Four-micrometer-thick sections were deparaffinized in xylene, stained with H&E, and then examined by light microscopy. To analyze histopathological features, especially those in the early stage of NASH, liver histopathological findings were scored using the validated system proposed by the NASH Clinical Research Network^3^ (Table 1), which we subdivided and modified.

To evaluate the presence of hemosiderin, which is the storage form of recycled iron in the liver and spleen, staining with Berlin blue, which is a ferric ferrocyanide produced by the reaction between ferric ions and ferrocyanide ions, was performed by a conventional method. The paraffin sections were stained with a mixed solution of hydrochloric acid and potassium ferrocyanide. After washing with distilled water, the sections were counterstained with Kernechtrot dye and dehydrated with alcohol. As shown in Supplementary Fig. S7, photographs of liver and splenic red pulp were taken in five randomly selected regions of interest (ROI) per sample, and the setting of arbitrary threshold in Berlin-blue-stained areas and quantification of such areas were performed using cellSens software (Olympus Co., Ltd., Tokyo, Japan). The ratio of a Berlin-blue-stained area was calculated as the average of the ratios of Berlin-blue-stained areas to ROI areas in five regions. The calculated values were robust against the magnification of a microscope because there was a positively significant correlation of the ratio of a Berlin-blue-stained area in low-magnification images with that in high-magnification images (Supplementary Fig. S8). The neutral lipid content in liver was determined by oil red O staining of frozen liver sections.

### Measurement of splenic iron level by inductively coupled plasma mass spectrometry (ICP-MS)

Iron level was measured by a previously described method^31^. Briefly, the mouse spleen was dispersed in ice-cold PBS and centrifuged at 500 × *g* for 20 min in 37% Percoll. After discarding the supernatant, red blood cell (RBC) lysis buffer was added to the pellet and incubated on ice. The suspension was centrifuged at 500 × *g* for 5 min and the resulting pellet was washed with RPMI medium twice. After the final centrifugation, the pellet was suspended in RPMI medium and splenic cells were counted using a Countess automated cell counter (Invitrogen, MA, USA). Splenic cells (5 × 10^6^ cells) in an aliquot of the medium was digested in ultrapure HNO_3_ and incubated overnight at room temperature. The digested cells were diluted in ultrapure water (1 : 20) and examined by ICP-MS (X-Series II; ThermoFisher Scientific, Inc., Waltham, MA, USA). The elementary standard for calibration was purchased from Kanto Chemical Co., Inc. (Tokyo, Japan). Iron level was compensated for by the protein level in splenic cells measured using a BCA protein assay kit (Pierce, IL, USA).

### Statistical analysis

The results were expressed as means ± standard error. Statistical analysis was performed using analysis of variance (ANOVA) followed by Fisher’s protect least significant difference (PLSD) test for multiple comparisons and Mann-Whitney’s U-test for nonparametric comparison with Stat View 5.0 (SAS, NC, USA). The strength of correlation between two variables was analyzed by Pearson’s correlation coefficient. Differences with a probability of 5% or less were considered significant.

## Additional Information

**How to cite this article**: Murotomi, K. *et al*. Involvement of splenic iron accumulation in the development of nonalcoholic steatohepatitis in Tsumura Suzuki Obese Diabetes mice. *Sci. Rep.* **6**, 22476; doi: 10.1038/srep22476 (2016).

## Supplementary Material

Supplementary Information
