# Supplementary material for: Involvement of splenic iron accumulation in the development of nonalcoholic steatohepatitis in Tsumura Suzuki Obese Diabetes mice
Source: Sci Rep. 2016 Mar 2;6:22476. doi: 10.1038/srep22476 (PMC4773882; doi:10.1038/srep22476)
Supplement: Supplementary Information [file srep22476-s1.pdf]

Supplementary Information

Involvement of splenic iron accumulation in the development of nonalcoholic steatohepatitis in Tumura Suzuki Obese Diabetes mice

Kazutoshi Murotomi[1*], Shigeyuki Arai[2*], Satoko Uchida[2], Shin Endo[2], Hitoshi Mitsuzumi[2], Yosuke Tabei[1], Yasukazu Yoshida[1], Yoshihiro Nakajima[1]

[1]Health Research Institute, National Institute of Advanced Industrial Science and Technology (AIST), Takamatsu, Kagawa 761-0395, Japan

[2]Hayashibara Co., Ltd., Naka-ku, Okayama 702-8006, Japan

*Two corresponding and first authors:

E-mail: k-murotomi@aist.go.jp (KM)

E-mail: shigeyuki.arai@hb.nagase.co.jp (SA)

Supplementary Figure S1

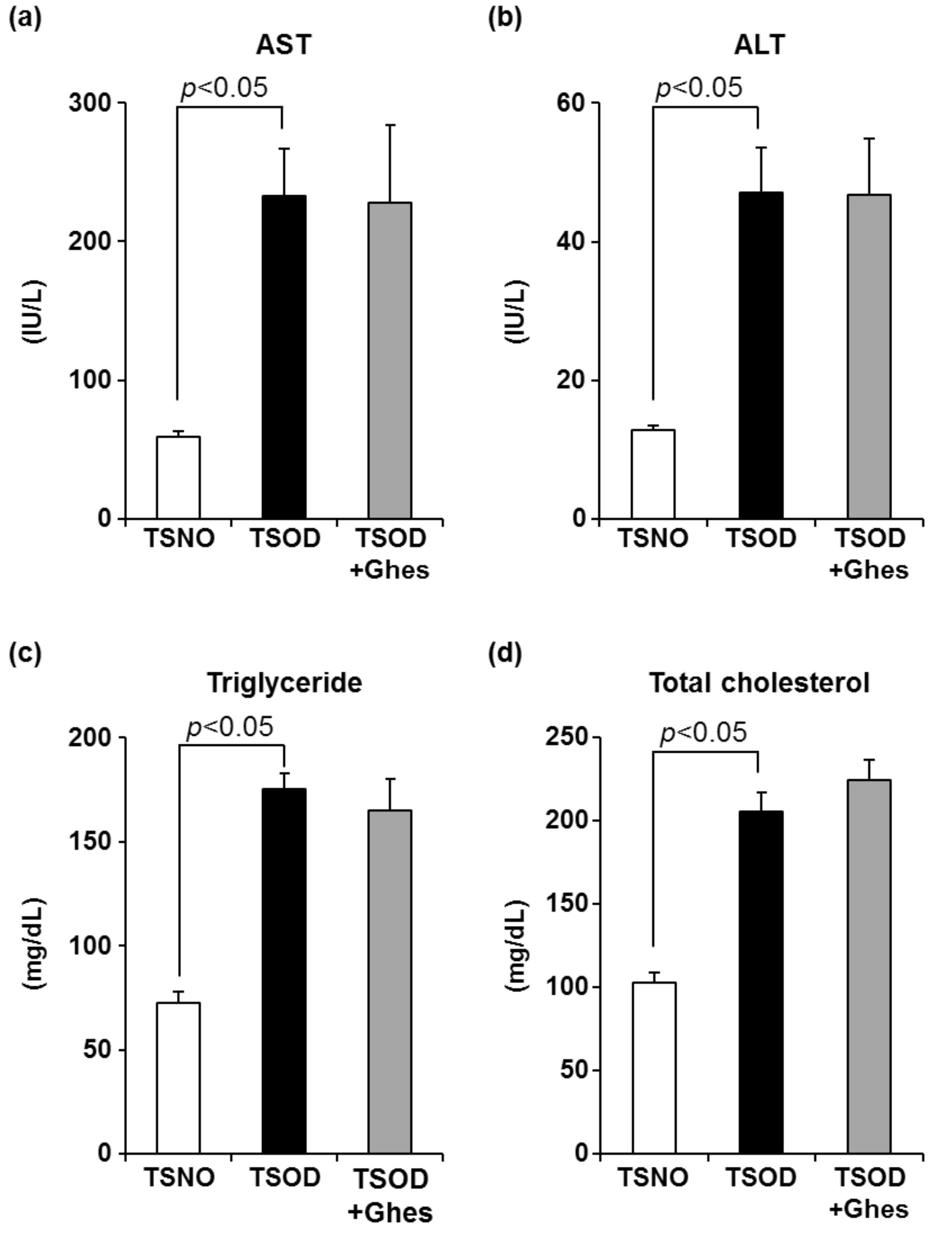

Figure S1 Measurement of serum biochemical parameters in TSNO, TSOD, and Ghes-fed TSOD mice.

AST (a), ALT (b), TG (c), and total cholesterol (d) levels in TSNO, TSOD, and Ghes-fed TSOD mice at 12 weeks of age. Results are expressed as means ± standard error (n = 6 each). Results were statistically evaluated by ANOVA (Fisher's PLSD test).

Supplementary Figure S2

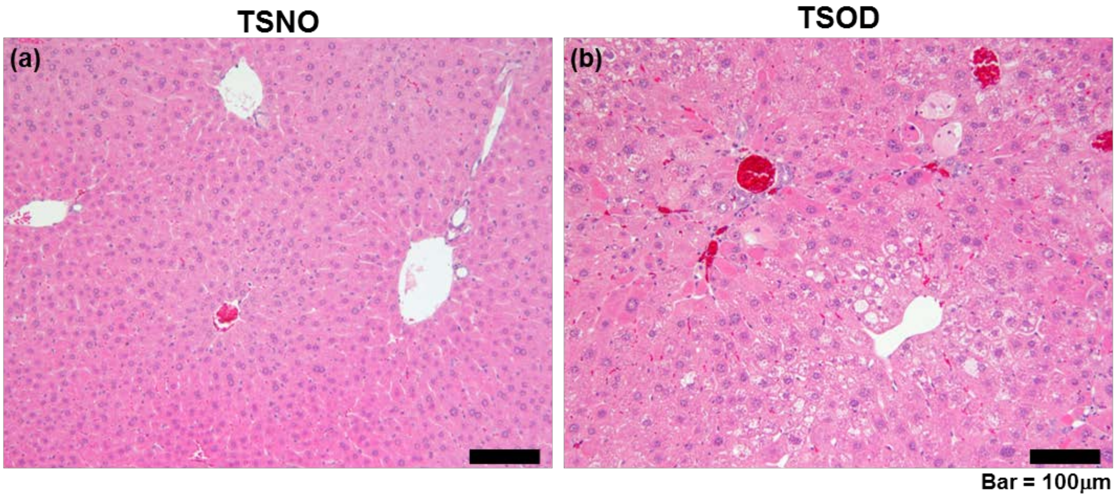

Figure S2 Characteristic liver histopathological findings in TSOD mice compared with TSNO mice at 12 weeks of age.

H&E-stained hepatic lobule sections from TSNO (a) and TSOD (b) mice (200 × magnification).

Supplementary Figure S3

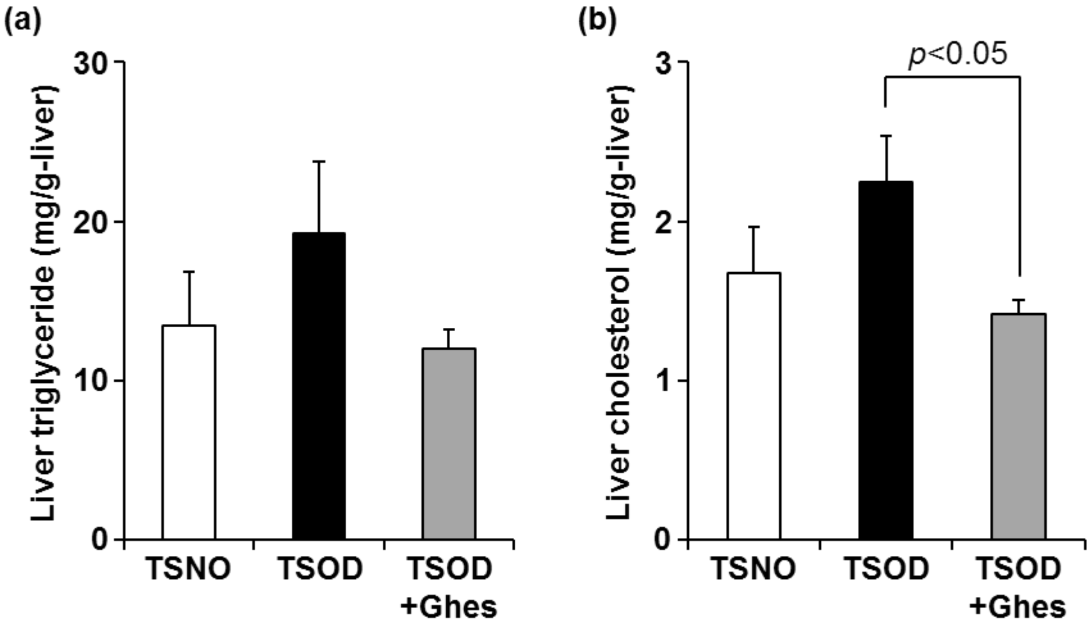

Figure S3 Comparison of hepatic lipid accumulation between TSOD and Ghes-fed TSOD mice.

Liver triglyceride (a) and cholesterol (b) levels in TSNO, TSOD, and Ghes-fed TSOD mice at 12 weeks of age. Results are expressed as means ± standard error (n = 6 each). Results were statistically evaluated by ANOVA (Fisher's PLSD test).

Supplementary Figure S4

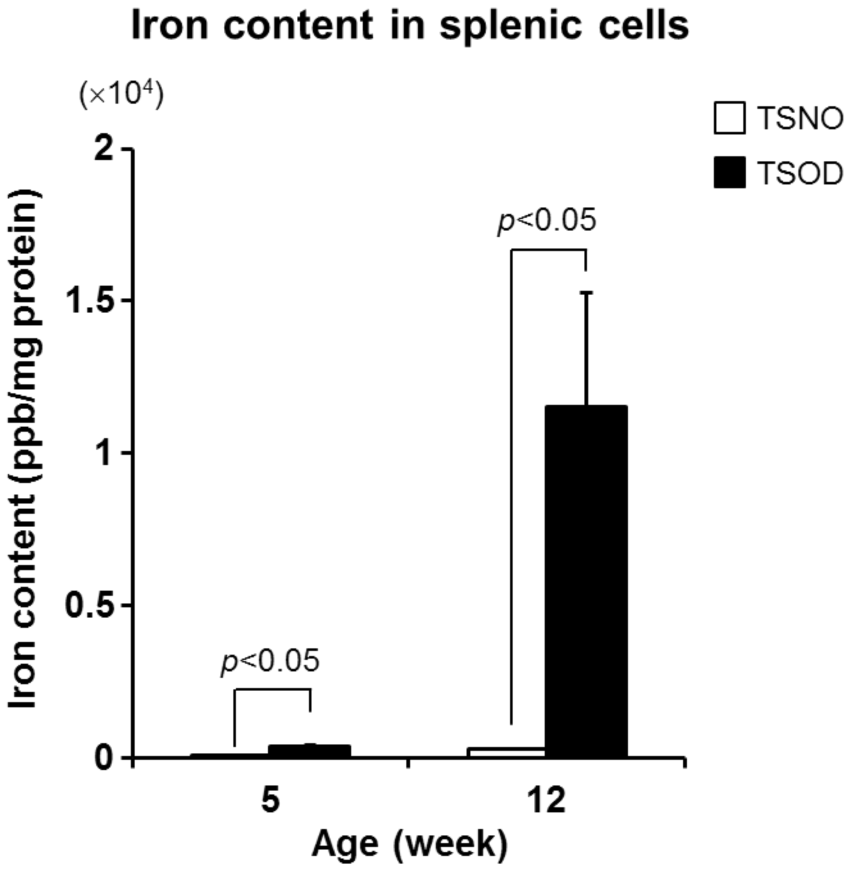

Figure S4 Analysis of splenic iron levels in TSNO and TSOD mice at 5 and 12 weeks of age with ICP-MS.

The iron level in splenic cells was measured by ICP-MS. An iron standard solution was used as reference and adjusted by the protein level in splenic cells. Results are expressed as means ± standard error (n = 5 each). Results were statistically evaluated by ANOVA (Fisher's PLSD test).

Supplementary Figure S5

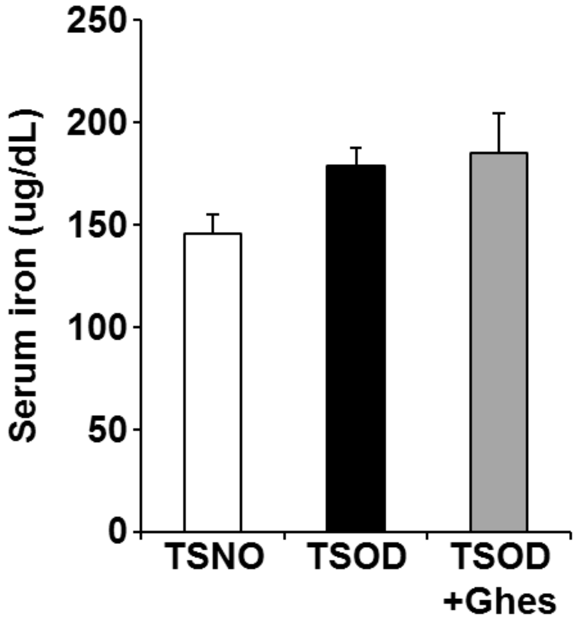

Figure S5 Measurement of serum iron levels in TSNO, TSOD, and Ghes-fed TSOD mice.

Serum iron levels in TSNO, TSOD, and Ghes-fed TSOD mice at 12 weeks of age. Results are expressed as means ± standard error (n = 6 each). There were no significant differences among the three groups.

Supplementary Figure S6

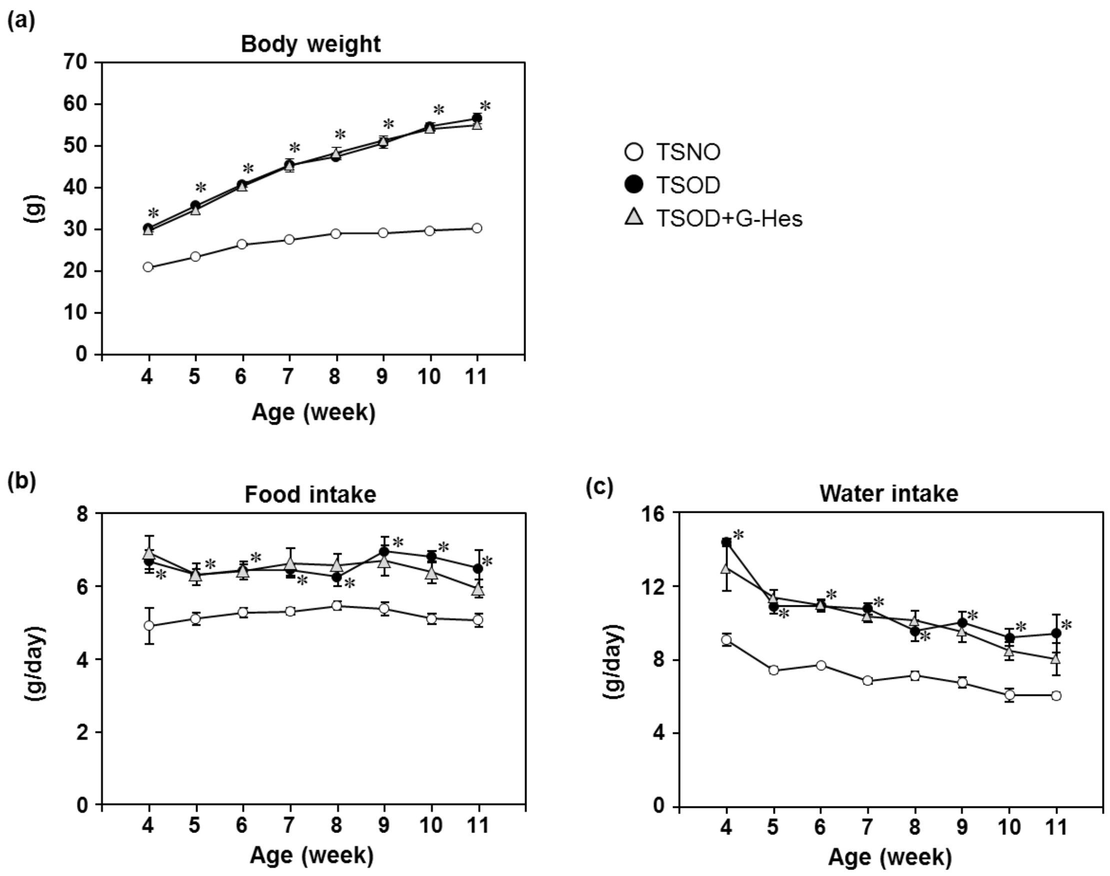

Figure S6 Changes in body weight, food intake, and water consumption in mice during experimental period.

The body weight (a), food intake (b), and water consumption (c) in TSOD mice were larger than those in age-matched TSNO mice. No significant differences in body weight (a), food intake (b), and water intake (c) between TSOD and Ghes-fed TSOD mice were observed throughout the experiments. Results are expressed as means ± standard error (n = 6 each). Results were statistically evaluated by ANOVA (Fisher's PLSD test). ∗Significantly different from TSNO mice ($p < 0.05$).

Supplementary Figure S7

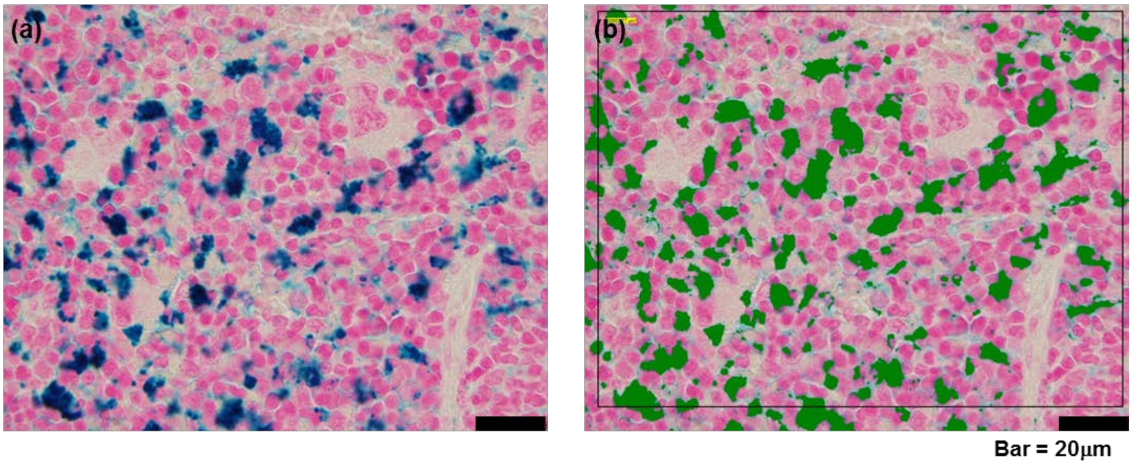

Figure S7 Representative ROIs used to calculate Berlin-blue-stained area.

Five ROIs (average area is 2000 μm^2) in the liver and spleen per sample were randomly selected. The thresholds in Berlin-blue-stained areas were arbitrarily set and areas were quantified using cellSens software (Olympus Co., Ltd., Tokyo, Japan) as shown in the above images.

Supplementary Figure S8

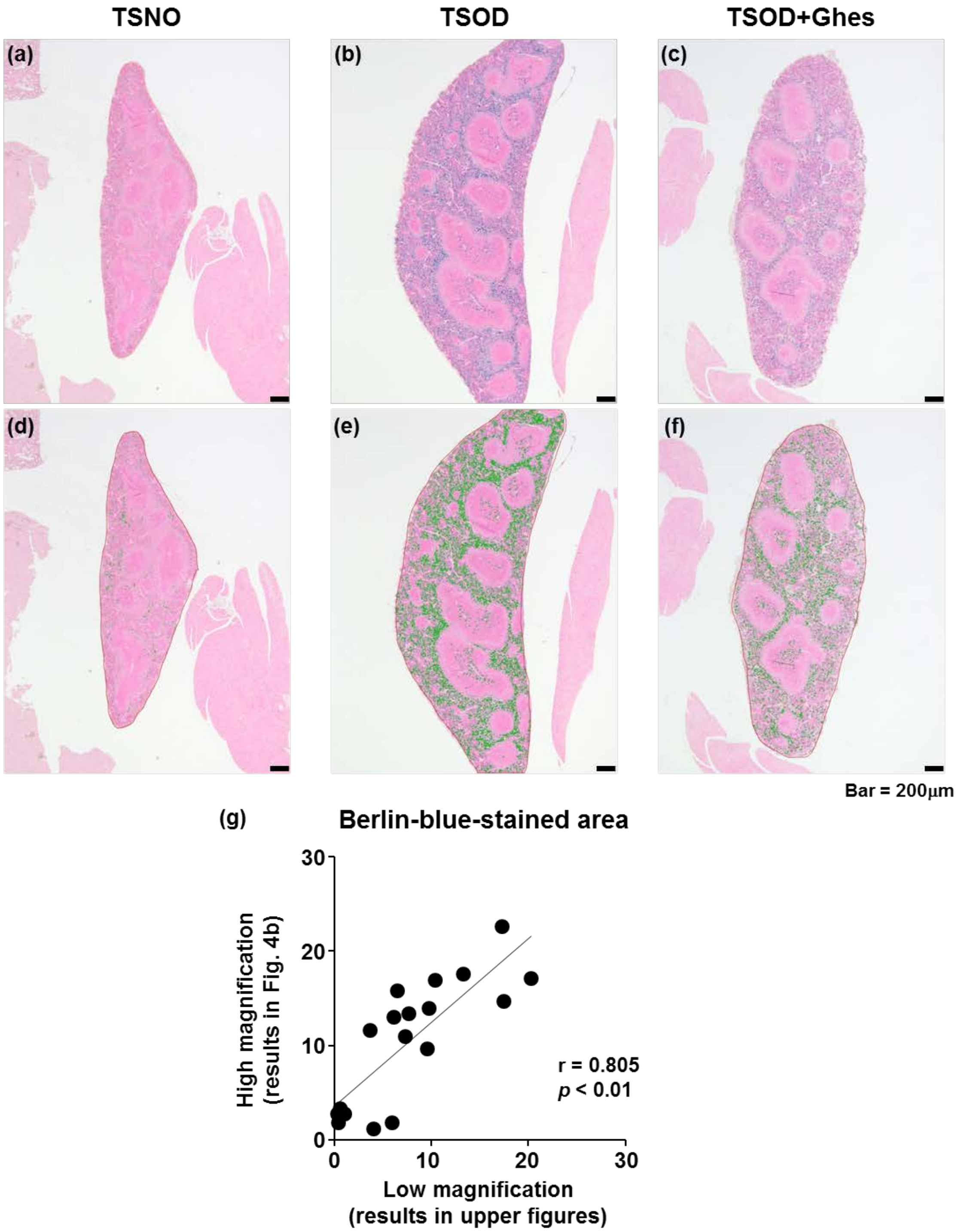

Figure S8 Analysis of correlation between low- and high-magnification images of spleen.

Representative findings of Berlin-blue-stained spleen sections from TSNO (a and d), control-diet-fed TSOD (b and e), and Ghes-fed TSOD (c and f) mice (40 × magnification). Berlin-blue-stained areas were quantified using cellSens software (Olympus Co., Ltd., Tokyo, Japan) and shown in green (d-f). Longitudinal axis indicates the ratio of Berlin-blue-stained area in high-magnification images (results in Fig. 4b) and horizontal axis indicates the ratio of Berlin-blue-stained area in low-magnification images (results in Supplementary Fig. S7), which was calculated as the ratio of a Berlin-blue-stained area to the total area of the spleen (g). The strength of the association between two parameters was evaluated on the basis of Pearson’s correlation coefficient.

Supplementary Table S1

Item	Definition	Score / Code
**Steatosis**		
Grade	Low- to medium-power evaluation of involvement by steatosis	
	< 5%	0
	5 - 33%	1
	> 33 - 66%	2
	> 66%	3
Location	Predominant distribution pattern	
	Zone 3	0
	Zone 1	1
	Azonal	2
	Panacinar	3
Microvesicular steatosis	Contiguous patches	
	Absent	0
	Rare	1
	Moderate	2
	Many	3
**Fibrosis**		
Stage		
	None	0
	Perisinusoidal or periportal	1
	Mild, zone 3, perisinusoidal	1A
	Moderate, zone 3, perisinusoidal	1B
	Portal / periportal	1C
	Perisinusoidal and portal / periportal	2
	Bridging fibrosis	3
	Cirrhosis	4
**Inflammation**		
Lobular inflammation	Overall assessment of all inflammatory foci	
	No foci	0
	< 2 foci per 100x field	1
	2 - 4 foci per 100x field	2
	> 4 foci per 100x field	3
Microgranulomas	Small aggregates of macrophages	
	Absent	0
	Rare	1
	Moderate	2
	Many	3
Large lipogranulomas	Usually in portal areas or adjacent to central veins	
	Absent	0
	Rare	1
	Moderate	2
	Many	3
Portal inflammation	Assessed at low magnification	
	Absent	0
	Rare	1
	Moderate	2
	Many	3
**Liver cell injury**		
Ballooning		
	Absent	0
	Rare	1
	Moderate	2
	Many	3
Acidophil bodies		
	Absent	0
	Rare	1
	Moderate	2
	Many	3
Pigmented macrophages		
	Absent	0
	Rare	1
	Moderate	2
	Many	3
Megamitochondria		
	Absent	0
	Rare	1
	Moderate	2
	Many	3
**Other findings**		
Mallory's hyaline	Visible on routine stains	
	Absent	0
	Rare	1
	Moderate	2
	Many	3
Glycogenated nuclei	Contiguous patches	
	Absent	0
	Rare	1
	Moderate	2
	Many	3

Table 1 Modified version of scoring based on NASH Clinical Research Network Scoring System Definitions
